# Apatinib in Advanced Gastric Cancer: A Doubtful Step Forward

**DOI:** 10.1200/JCO.2016.68.6931

**Published:** 2016-08-15

**Authors:** Lorenzo Fornaro, Enrico Vasile, Alfredo Falcone

**Affiliations:** Lorenzo Fornaro, Enrico Vasile, and Alfredo Falcone, Azienda Ospedaliero-Universitaria Pisa, Pisa, Italy

## To the Editor:

Li et al^1^ have recently reported the results of a phase III trial that evaluated apatinib treatment in patients with advanced gastric cancer who experienced disease progression after two or more lines of systemic therapy. The authors compared apatinib—a tyrosine kinase inhibitor that targets the vascular endothelial growth factor receptor-2 (VEGFR-2)—with placebo among 273 randomly assigned patients and reported an overall survival advantage in favor of the experimental treatment. This study represents an important step forward in gastric cancer research, as it confirms the value of targeting VEGFR-2 beyond the already available evidence that supports the use of ramucirumab, an anti-VEGFR-2 antibody, in the second-line setting.^2,3^ However, there are several potential pitfalls that may limit the value of these results.

With regard to patient characteristics, an imbalance in performance status (PS) distribution is evident, with an excess of patients with a PS of 0 in the experimental arm compared with placebo (27.3% *v* 16.5%). The authors conclude that this difference does not formally reach statistical significance, but, in our opinion, this could have had an unpredictable impact on overall survival. Indeed, PS is one of the main determinants of survival in advanced gastric cancer from first-line to third-line setting,^4-6^ and even a seemingly mild deterioration in the general condition, that is, a PS of 1 according to the Eastern Cooperative Oncology Group scale, might impair outcomes in such a fragile patient scenario.

In terms of safety, apatinib resulted in a not negligible rate of grade 3 to 4 hand-foot syndrome (8.5%), with approximately one of two patients experiencing proteinuria (generally grade 1 to 2) and 5.7% of patients experiencing grade 3 to 4 neutropenia. Is it really reasonable to conclude that apatinib “has a favorable safety profile in comparison with other antiangiogenic agents?”^1(p1452)^ In the larger REGARD trial, single-agent ramucirumab resulted in a much lower rate of proteinuria (all grade, 4%), and grade 3 to 4 neutropenia and hand-foot syndrome were not reported.^2^ Whereas these adverse events are manageable when apatinib is administered as monotherapy, they may not suit well when apatinib is combined with cytotoxic agents used in gastric cancer, such as fluoropyrimidines and platinum compounds. With regard to cardiac safety, initial data with apatinib seems reassuring; however, comparisons with sunitinib and bevacizumab—as reported in the Discussion of the article by Li et al^1^ and for which we have greater knowledge about potentially severe cardiovascular events—seem at least premature. This is particularly true if we keep in mind that elderly patients (age > 70 years) were excluded from the trial, the number of patients between age 65 and 70 years was limited (37 patients), and the median age in the two arms (age 58 years) is lower than that observed in routine practice.^1^ In the RAINBOW trial with ramucirumab, despite a similar median age in the enrolled patient cohorts, the upper age limit was 84 years and the number of patients age ≥ 65 years was greater (249 patients). Neither in the REGARD trial^7^ nor in the RAINBOW trial^8^ did the age subgroup analyses report increased toxicity among older patients, with the exception of a relatively higher rate of grade ≥ 3 neutropenia in the RAINBOW trial.

Finally, no significant improvement in quality of life was noted for apatinib.^1^ Quality-of-life data for ramucirumab monotherapy have been recently published; ramucirumab proved to delay the worsening of symptoms and the deterioration of PS.^9^ It should be recognized that the population enrolled in the apatinib trial had a far more pretreated disease, but patient characteristics of PS, age, and number of metastatic sites seem comparable between trials.^1,2^

With an arguably increasing number of patients receiving ramucirumab in the second-line setting, it is difficult to anticipate the potential impact of apatinib in routine clinical practice. As these agents share the same target along the pathways that regulate tumor angiogenesis, the efficacy of apatinib in overcoming resistance to ramucirumab is unclear. Moreover, results with apatinib have been reported among Chinese patients. As a result of differences in the biologic background and treatment patterns between Eastern and Western patients with gastric cancer,^10^ other studies should be awaited to confirm these data outside Asia.

To conclude, apatinib is another arrow in the bow against gastric cancer. Future trials and, we hope, the identification of predictive biomarkers will ultimately tell us how far we can hurl our hope in this challenging struggle.
